# Doppler Radar Sensor-Based Fall Detection Using a Convolutional Bidirectional Long Short-Term Memory Model

**DOI:** 10.3390/s24165365

**Published:** 2024-08-20

**Authors:** Zhikun Li, Jiajun Du, Baofeng Zhu, Stephen E. Greenwald, Lisheng Xu, Yudong Yao, Nan Bao

**Affiliations:** 1 The College of Medicine and Biological Information Engineering, Northeastern University, Shenyang 110167, China; 2371307@stu.neu.edu.cn (Z.L.); 2171230@stu.neu.edu.cn (J.D.); xuls@bmie.neu.edu.cn (L.X.);; 2 The School of Computer Science and Engineering, Northeastern University & Neusoft Research of Intelligent Healthcare Technology, Shenyang 110167, China; zhubaofeng@stumail.neu.edu.cn; 3 The Blizard Institute, Barts & The London School of Medicine & Dentistry, Queen Mary University of London, London E1 4NS, UK; s.e.greenwald@qmul.ac.uk

**Keywords:** fall detection, deep learning, convolutional neural network, bidirectional long short-term memory, doppler radar, temporal sequential feature, spatial feature

## Abstract

Falls among the elderly are a common and serious health risk that can lead to physical injuries and other complications. To promptly detect and respond to fall events, radar-based fall detection systems have gained widespread attention. In this paper, a deep learning model is proposed based on the frequency spectrum of radar signals, called the convolutional bidirectional long short-term memory (CB-LSTM) model. The introduction of the CB-LSTM model enables the fall detection system to capture both temporal sequential and spatial features simultaneously, thereby enhancing the accuracy and reliability of the detection. Extensive comparison experiments demonstrate that our model achieves an accuracy of 98.83% in detecting falls, surpassing other relevant methods currently available. In summary, this study provides effective technical support using the frequency spectrum and deep learning methods to monitor falls among the elderly through the design and experimental validation of a radar-based fall detection system, which has great potential for improving quality of life for the elderly and providing timely rescue measures.

## 1. Introduction

Currently, indoor fall accidents have become one of the leading causes of non-disease-related deaths among the elderly [1]. According to data from the National Institutes of Health in the United States, approximately 1.6 million elderly people are affected by fall-related injuries each year [2]. At the same time, the elderly population is rapidly increasing worldwide. China is facing the greatest rate of population aging in human history, with around 17% of the population being over sixty years old in 2020. By 2050, this proportion is expected to rise to approximately 35% [2]. To prevent the frequent occurrence of such events, it is crucial to design a robust and reliable fall detection system.

Currently, there are various tools used for fall detection among the elderly, such as wearable sensors [3] and video monitoring systems [4]. However, these tools have certain limitations. Wearable sensors provide timely feedback on the body’s signals, but they must be worn constantly and require regular battery replacement [5]. Video monitoring systems, while providing much information, are susceptible to obstruction by objects and raise privacy concerns [6]. Wireless-signal-based detection systems, on the other hand, offer advantages such as small size, low power consumption, easy deployment without the need for wearing, and resistance to environmental interference [7,8]. Common types of wireless-signal-based devices include Wi-Fi- [9], infrared- [10], and radar-based systems [11]. Infrared signals must be protected from changing environmental factors such as lighting conditions, which can be challenging in practical applications [12]. Wi-Fi signals are prone to overlapping with regular communication channels, resulting in unwanted interference and even the collection of users’ private information. In comparison, radar-signal-based systems are stable and unaffected by factors such as dust, lighting conditions, and nearby objects [13]. These factors make radar technology more effective for fall detection and motion recognition in general.

In the field of radar-based fall detection, there have been several previous reports. For example, He, M. et al. [14] used a support vector machine approach to extract features from radar spectrogram data. Wang, B. et al. [15] used the line kernel convolutional neural network (LKCNN) to extract spatial features from spectrograms. Trange, A. [16] treated the spectrogram as a temporal sequential signal and processed it using conventional long short-term memory (LSTM). Other approaches include that of Anishchenko, L. et al. [17], who used two bioradar devices. The bioradar with a wavelength of 24.107 GHz can capture the characteristics of falls. Feng, X. et al. [18] utilized multiple radar devices to generate spectrogram matrices for analysis. The existing methods for radar feature extraction have certain limitations, such as low accuracy, the inability to capture enough effective features of the radar spectrogram, or the use of multiple radar devices for data collection, resulting in increased costs. Thus, proposing a more accurate fall detection method on a Doppler radar sensor remains a challenge.

Motivated by these insights, we developed a deep-learning-based fall detection network on a Doppler radar sensor called convolutional bidirectional long short-term memory (CB-LSTM), consisting of a convolutional neural network (CNN) and a bidirectional long short-term memory (BiLSTM). We collected a large number of different types of fall and non-fall activities, and the proposed model was trained and verified through extensive experiments.

To summarize, our work and contributions are as follows:

- The proposed deep learning model CB-LSTM utilizes the CNN and BiLSTM network architectures, extracting the spatial features and temporal sequential features of the radar frequency spectrum, respectively, enhancing the accuracy and reliability of the detection.
- In order to make the fall data used here closer to daily life, we comprehensively simulated various fall states, where the non-fall data consisted of everyday activities that are easily confused with falling.
- Extensive experiments were conducted to evaluate the performance of our proposed method. The results of the ablation experiments and comparative experiments demonstrated that our proposed CB-LSTM model achieved good fall detection accuracy, providing effective technical support for the preventing falls among the elderly.

The remainder of this paper is organized as follows: Section 2 introduces the process of data processing, including radar signal, radar frequency spectrogram, and signal denoising. In Section 3, we propose and introduce the CB-LSTM model for fall detection. In Section 4, the experimental setup is introduced. Section 5 sets out the experimental results. Section 6 discusses some limitations and future works, and, finally, Section 7 concludes this paper.

## 2. Data Processing

### 2.1. Radar Signal

Continuous-wave (CW) radar has long been one of the preferred radar technologies for observing human motion and is relatively simple to implement. It operates by transmitting a continuous signal at a fixed frequency and receiving the mixed signal of the reflected object and the transmitted carrier signal [19]. This allows the detection of the object’s radial velocity changes through the Doppler effect [20]. When used to detect body movement, the Doppler shift carries information about the velocity of various body parts, including the torso and limbs.

However, non-modulated CW radar can only monitor the velocity of objects and cannot measure the distance between the target and the transmitter. In contrast, broadband radar systems, such as frequency modulated continuous-wave (FMCW) radar, can overcome this limitation [21]. FMCW radar emits a signal with a continuously varying frequency, allowing it to measure both the range and velocity of objects [22]. This type of radar offers higher measurement accuracy and has found wide applications in diverse fields.

In radar distance measurement systems, the empirical radar range equation (RRE) in (1) is commonly used as the foundation.

(1) $$R={\left[\frac{P_{t}\lambda^{2}G_{t}G_{r}\sigma }{{\left(4\pi \right)}^{3}P_{r}L_{f}L}\right]}^{1/4},$$

where *R* represents the distance between the target and the radar transmitter. *P_t_* is the transmission power of the CW radar, measured in watts. *G_t_* is the gain of the radar transmit antenna. *G_r_* is the gain of the radar receive antenna. *λ* is the operating wavelength of the radar, measured in meters. *σ* is the radar cross-section of the target, measured in square meters. *P_r_* is the received power at a distance *R*. *L_f_* is the correction factor for losses caused by fluctuations in the radar cross-section of the target. *L* is the loss factor of the radar system, including transmission losses, reception losses, and others.

### 2.2. Radar Frequency Spectrogram

In traditional approaches, to reflect the relationship between velocity and Doppler frequency, time–frequency (TF) analysis methods are commonly used to represent the backscattered signals from moving subjects [23,24]. By analyzing the signals in both the time and frequency domains, the time-varying characteristics of body movement and the variations in Doppler frequency can be better revealed, allowing for more effective processing and signal analysis. The most commonly used method in TF signal representation is to transform the signal into a spectrogram because this provides an intuitive depiction of the power distribution of the signal over time and frequency [25]. Specifically, a spectrogram transforms the signal from the time domain to the frequency domain, visualizing the power distribution of the signal over a range of frequencies and how this changes with time. The spectrogram of a discrete signal is given by Equation (2).

(2) $$\mathrm{SPEC}(n,k)={\left|\sum_{m=0}^{N-1}w(m)s(n-m)e^{-j2\pi km/N}\right|}^{2},$$

where SPEC(*n*, *k*) represents the complex value of the k-th frequency component in the frequency domain. It is the result of computing the discrete Fourier transform (DFT) of the discrete signal sequence x[*n*] of length *N*, i.e., the total number of samples. The variable *n* represents the index of the sample point in the time domain, ranging from 0 to *N* − 1. The variable m is used for summation in the equation and takes values in the range of 0 to *N* − 1. w(*m*) is a weighting coefficient that is applied to each sample point, typically given by *e^−j2km/N^*. s(*n* − *m*) represents the sample point of the signal sequence x[*n*] at the time domain index *n* to m. *e^−j2km/N^* is the complex exponential term that describes the frequency component in the frequency domain, where *k* is the index of the frequency point in the frequency domain.

### 2.3. Signal Denoising

When a radar signal is transmitted, stationary objects in the beam path cause reflections, sometimes known as ground clutter. These signals can mask the low-frequency components of moving targets in the derived spectrum, affecting the reliability and accuracy of the Doppler-shifted reflections from moving objects [26]. Therefore, to accurately detect and measure moving targets, appropriate signal processing methods must be employed to reduce the impact of ground clutter.

**Figure 1:**
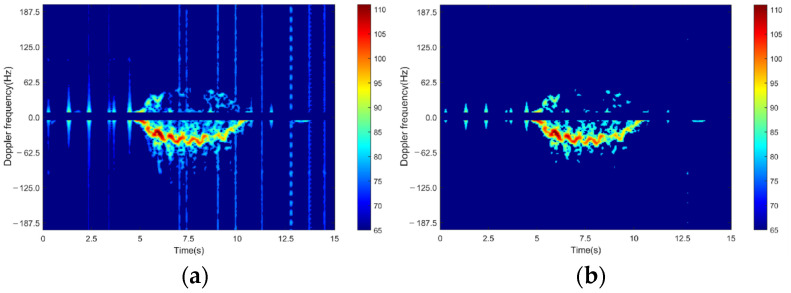
Spectrogram denoising: (**a**) original spectrogram; (**b**) spectrogram processed with MTI.

One effective technique is the employment of moving target indication (MTI) technology. MTI separates moving targets from ground clutter by calculating the difference between target motion and ground clutter and suppressing it before the frequency spectrum is generated [27]. Figure 1 shows the appearance of an original spectrogram and a spectrogram processed with MTI. The spectrogram size used in this experiment was 205 × 450, where 205 represents the frequency range of radar acquisition, and 450 is the number of frames acquired in 15 s at a rate of 30 Hz. The color scale in the figure represents signal intensity, measured in dB.

## 3. Proposed Model for Fall Detection

### 3.1. CB-LSTM Model

**Figure 2:**
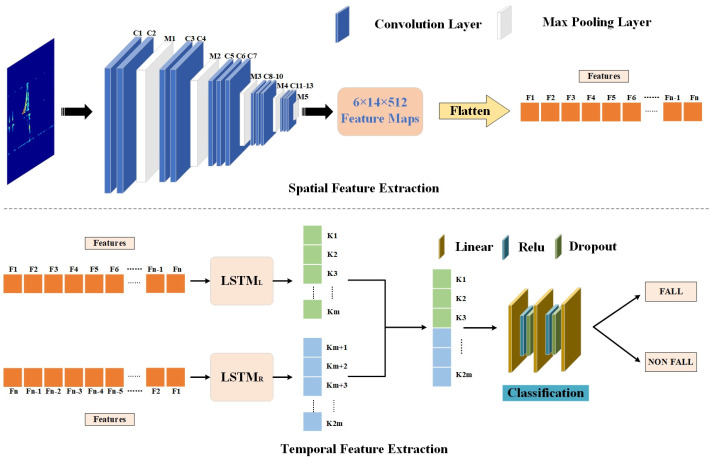
CB-LSTM architecture. LSTM_L_ and LSTM_R_ represent the forward LSTM and backward LSTM, respectively; C*n* represents the *n*-th convolutional layer; M*n* represents the *n*-th max pooling layer.

We propose a deep learning network called CB-LSTM that combines a CNN [28] and BiLSTM [29] for fall detection based on radar signals. CNNs are typically used for extracting spatial features from data, while BiLSTM, a variant of the recurrent neural network (RNN), is commonly employed for handling time-series-related problems. In the CB-LSTM model, the CNN is responsible for extracting spatial and feature information from the radar frequency spectrum. The BiLSTM network, on the other hand, focuses on learning the temporal sequential dependencies and patterns within the radar signal. By combining the strengths of both the CNN and BiLSTM, the CB-LSTM model aims to improve the accuracy and robustness of fall detection. Figure 2 and Table 1 show the structural framework of the CB-LSTM model. This hybrid architecture allows the CB-LSTM model to effectively capture both the spatial and temporal sequential features of the radar signals, enabling it to make more accurate and reliable predictions in target detection tasks.

Firstly, we employ the CNN model to extract spatial features from the spectrogram. The feature extraction part consists of convolutional layers and pooling layers, followed by a classifier composed of linear layers, the ReLU activation function, and a SoftMax layer. This efficient architecture improves the accuracy of the model. By loading pretrained CNN weights (trained on ImageNet) into the model for the new task, we can significantly enhance the accuracy and increase the convergence speed. To enhance the feature extraction stage, the original input image size is set to 205 × 450. Convolutional layers and pooling layers with channel numbers of 64, 128, 256, and 512 are applied successively to gradually reduce the input image size. This process results in high-dimensional features of size 6 × 14 × 512.

Next, the high-dimensional features are flattened into a 1 × 1 × 43,008 one-dimensional vector, denoted as F1, F2, …, Fn, where the data dimension and batch size are both 1, and the data length *n* is 43,008. This procedure allows for easy input into the BiLSTM network. The main component of this network is the LSTM [30], which evolved from the classical RNN. Compared to an RNN, LSTM can more effectively handle sequence data and leverage long-range dependencies within the input sequence. This helps to overcome the problems of vanishing or exploding gradients and allows for the rapid capture of important features within the sequence [31]. BiLSTM consists of a forward LSTM and a backward LSTM, each having its own hidden state and cell state. In the forward LSTM, the features are input in the order of F1, F2, …, Fn, and the hidden state and cell state information propagate from front to back. This process results in a one-dimensional vector [K1, K2, …, Km]. In the backward LSTM, the input sequence is reversed, while the hidden state and cell state information propagate from back to front, producing a vector [Km + 1, Km + 2, …, K2m]. As a result, the BiLSTM model can obtain a comprehensive feature representation at each time step, incorporating all the information from both the preceding and succeeding steps.

Then, the extracted bidirectional features are concatenated into a one-dimensional vector [K1, K2, …, Km, Km + 1, …, K2m], which serves as the input for the fully connected layer for classification. The fully connected layer used in this paper consists of multiple linear layers, ReLU functions, and dropout functions. In the fully connected layer, the linear layer performs a weighted summation of the inputs from the previous layer. As each neuron in the fully connected layer is connected to all neurons in the previous layer, this step captures the relationships between all the features. The ReLU function is an activation function commonly used in neural networks; it introduces nonlinearity to enhance the expressive power of the network, benefiting from its good convergence properties. The dropout function, a regularization technique commonly used in neural networks to prevent overfitting, is then applied to reduce the model’s complexity while improving its generalization ability. With this architecture, the fully connected layer can globally process the features extracted by the CNN and BiLSTM, capturing the global information and subsequently performing classification.

### 3.2. Optimizer and Training Parameters

The experiments in this study used a batch size of 1. We used the Adam optimizer, with an initial learning rate of 0.001. Training was stopped if the model did not improve its performance on the validation set after 100 epochs or if it had been running for 500 epochs. The experiments were conducted on an Intel(R) Xeon(R) Gold 5218 CPU and an RTX 3090 GPU. The development language was python. The hardware configuration was utilized to accelerate the training and testing processes of the classification model, significantly reducing the iteration time.

### 3.3. Loss Function

The loss function we use is binary cross-entropy (BCE), implemented with ‘nn.BCELoss(·)’, which calculates the loss based on the expression (3):

(3) $$L=-\frac{1}{N}\sum_{i=1}^{N}\left[y_{i}\log(p_{i})+(1-y_{i})\log(1-p_{i})\right],$$

where *N* represents the total number of samples, *y_i_* represents the class label of the *i*-th sample, and *p_i_*, the predicted value for the *i*-th sample. When training a binary classification model, it is customary to apply a sigmoid function to the model’s output, which maps the output to a probability value between 0 and 1.

### 3.4. Quantitative Evaluation

The model was evaluated using the accuracy, precision and recall metrics, as follows:

(4) $$\mathrm{Accuracy}=\frac{\mathrm{TP}+\mathrm{TN}}{\mathrm{TP}+\mathrm{TN}+\mathrm{FP}+\mathrm{FN}},$$

(5) $$\mathrm{Precision}=\frac{\mathrm{TP}}{\mathrm{TP}+\mathrm{FP}},$$

(6) $$\mathrm{Recall}=\frac{\mathrm{TP}}{\mathrm{TP}+\mathrm{FN}},$$

where TP represents true positive, TN represents true negative, FN represents false negative, and FP represents false positive.

## 4. Experimental Setup

The signal transmitter and receiver used in this study employed the CL2440 system-on-chip (SoC) radar module developed by Celeno Corporation, Ra’anana, Israel. The internal software was based on the Linux operating system.

**Figure 3:**
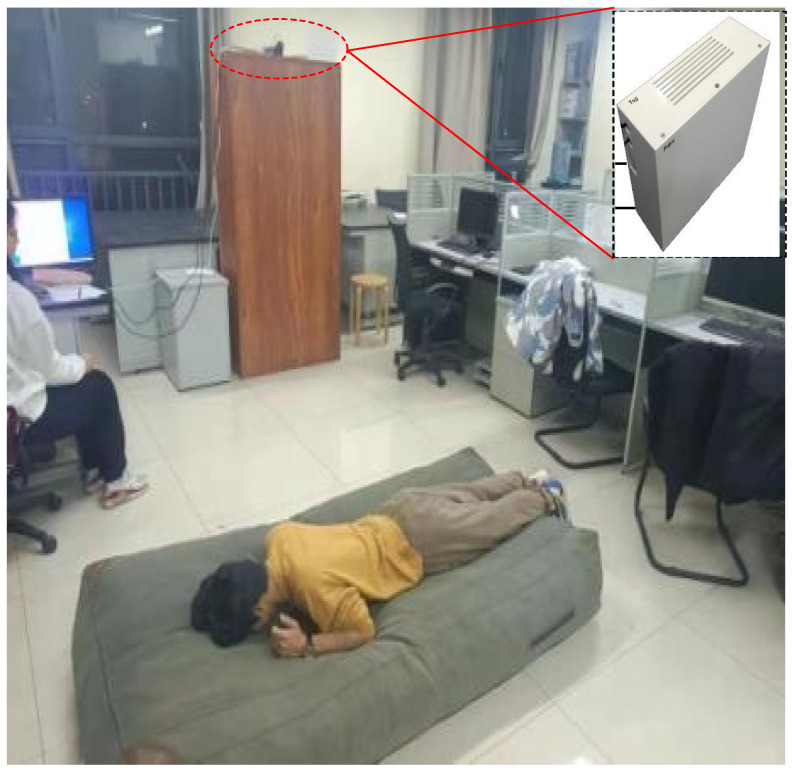
Experimental site. The radar system, circled in red, was positioned on the cabinet in the background and is shown enlarged in the inset.

As shown in Figure 3, the radar was positioned above a cabinet on one side of a room, at a height of approximately two meters above the floor, and with the tilt angle set to ensure maximum coverage of the experimental area. The experimental room was approximately 5 m by 8 m with a height of 3 m, and the volunteer testing area was approximately 3 m by 5 m with a height of 3 m.

**Figure 4:**
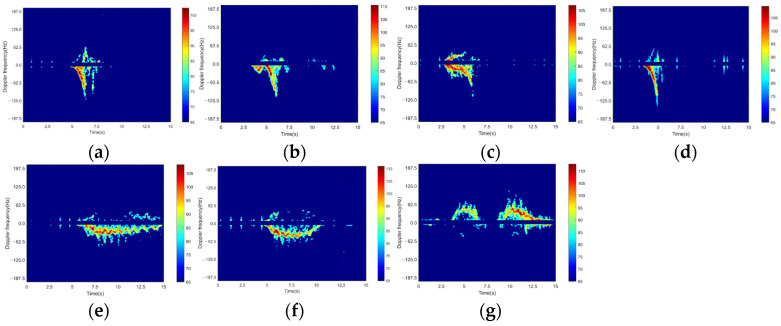
Examples of the different types of activities investigated: (**a**) direct fall; (**b**) kneel and fall; (**c**) walk and fall; (**d**) sit and fall; (**e**) walk; (**f**) walk and squat down; (**g**) walk, squat down, then stand up.

Altogether, 97 volunteers participated in the testing, all of whom were students aged 18–22, including 57 men. The total of 4435 data samples consisted of 2475 falls and 1960 non-falls. The non-fall data simulated common actions that could be confused with falls in daily life, such as walking, squatting down, then standing up. The fall data simulated several common types of falls, including direct fall, kneel and fall, walk and fall, and sit and fall. Table 2 displays the data quantity and the corresponding labels for each of the activities. These events are closer to those occurring in everyday life compared to typical datasets in other studies, especially the fall data, which cover a wider range of falling scenarios. Example spectrograms of different actions are shown in Figure 4.

## 5. Results

**Figure 5:**
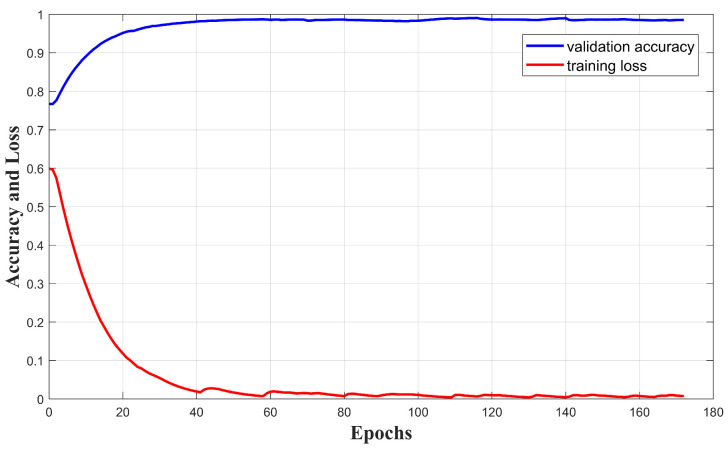
Accuracy and loss of CB-LSTM.

**Figure 6:**
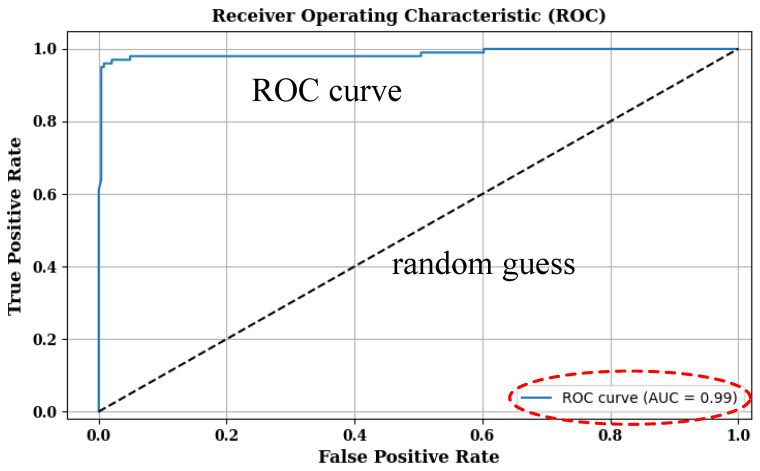
ROC curve.

The data collected were randomly allocated into training, validation, and test sets at a ratio of 8:1:1. Consequently, the variations in the model training and validation processes were plotted, as shown in Figure 5, where it can be seen that the validation accuracy is close to 1, and the loss curve initially has some fluctuations but eventually converges. The training loss and the validation accuracy tended to approach an unchanging value at around 70 epochs. The trained model was evaluated on the test set, yielding an accuracy of 0.9883, a precision of 0.9878, and a recall of 0.9918. We also conducted a receiver operating characteristic (ROC) analysis. As shown in Figure 6, the area under the ROC curve (AUC) is 0.99, indicating a very low false alarm rate and showing that the classification results of our model are reliable. These results demonstrate that the proposed model offers excellent performance in fall detection.

Our model is based on and developed from LSTM, and we therefore compared it to the general models, including LSTM and BiLSTM. The results of the comparison experiments are shown in Table 3. Compared to LSTM, the proposed fall detection model, which integrates CNN, improves the accuracy by 6.67%, precision by 9.46%, and recall by 13.99%. Compared to BiLSTM, the proposed model increases the accuracy by 4.22%, precision by 6.62%, and recall by 10.5%.

We also compared our approach with the state-of-the-art methods for radar-based fall detection. Table 4 shows the comparison results. It is clear that the novel structure of our model, CNN + BiLSTM, is highly effective as it extracts both spatial and temporal sequential features simultaneously, resulting in improved performance as a means of radar spectrum fall detection.

In addition, we conducted separate tests on each activity, and the results are shown in Table 5. It can be seen that the walking recognition rate is higher in non-fall scenarios, while the recognition accuracy of direct falling in fall activities is higher, since walking in non-fall and direct falling in fall are single and simple activities. In order to simulate real-life scenarios, other collected data activities are more complex and easily confused. However, our fall detection model also achieved a good recognition rate for these easily confused activities in fall detection.

## 6. Limitations and Future Work

This study achieved some good experimental results. However, there are still some limitations. Firstly, we simulated many different types of falls, but the diversity of the non-fall movements in daily life is limited, and the data volume is smaller than that of falls. Additionally, our experiment was conducted entirely indoors and consistently collected single-person actions, which poses certain limitations for real-world application scenarios. Despite the above limitations, we believe that this study is valid in fall detection research.

In the future, we will continue to collect more fall and non-fall movement data, especially by increasing the variety and quantity of daily movements. Then, we will use more test data to validate the performance and robustness of our proposed method. Secondly, we will attempt to collect outdoor data and data involving multiple individuals, broadening the applicability of the system in daily life.

## 7. Conclusions

This study proposed a deep learning model CB-LSTM for fall detection based on the Doppler radar frequency spectrum. To increase the accuracy and reliability of fall detection, the proposed model utilizes CNN and BiLSTM network architectures, extracting the spatial features and temporal sequential features of the radar frequency spectrum, respectively. In order to make the fall data used here closer to daily life, we comprehensively simulated various fall states, while the non-fall data consisted of everyday activities that are easily confused with falling, enhancing the robustness of the model. The experiment results demonstrate that our proposed CB-LSTM model offers good performance for fall detection, which can be used for intelligent monitoring of the elderly.

## Figures and Tables

**Table 1:** Detailed structure of CB-LSTM.

Layer Type	No. of Channels	Feature Map Size
Input	-	(205, 450)
Conv2d	64	(205, 450)
Conv2d	64	(205, 450)
Maxpool2d	64	(102, 225)
Conv2d	128	(102, 225)
Conv2d	128	(102, 225)
Maxpool2d	128	(51, 112)
Conv2d	256	(51, 112)
Conv2d	256	(51, 112)
Conv2d	256	(51, 112)
Maxpool2d	256	(25, 56)
Conv2d	512	(25, 56)
Conv2d	512	(25, 56)
Conv2d	512	(25, 56)
Maxpool2d	512	(12, 28)
Conv2d	512	(12, 28)
Conv2d	512	(12, 28)
Conv2d	512	(12, 28)
Maxpool2d	512	(6, 14)
Flatten	-	(1, 6 × 14 × 512)
BiLSTM	-	(1, 256 × 512)
FC	-	(1, 2)
Transpose	-	(2, 1)

**Table 2:** The quantity and labels of different actions.

Action Type	Quantity	Label
Walk	326	Non-fall
Walk and squat down	318	Non-fall
Walk, squat down, then stand up	326	Non-fall
Direct fall	656	Fall
Kneel and fall	619	Fall
Walk and fall	598	Fall
Sit and fall	602	Fall

**Table 3:** Comparison experiment with general models.

	Accuracy	Precision	Recall
LSTM	0.9216	0.8932	0.8519
BiLSTM	0.9461	0.9216	0.8868
CB-LSTM	0.9883	0.9878	0.9918

**Table 4:** Comparison experiment with SOTA methods.

	Accuracy	Precision	Recall
Wang, B. et al. [15]	0.9874	0.9755	0.9963
Sadreazami, H. et al. [32]	0.9583	0.9837	0.9437
Trange, A. [16]	0.9200	0.9400	0.8500
Jokanović, B. et al. [19]	0.9710	0.8795	0.8824
CB-LSTM	0.9883	0.9878	0.9918

**Table 5:** Accuracy of detecting different activities.

	Label	Accuracy	Average Accuracy
Walking	Non-fall	0.9913	0.9870
Walk followed by squatting down		0.9897	
Walk, squat down, then stand up		0.9799	
Direct fall	Fall	0.9949	0.9909
Falling from a kneeling position		0.9899	
Falling while walking		0.9917	
Falling from a sitting position		0.9871	

## Data Availability

The raw data supporting the conclusions of this article will be made available by the authors on request.

## References

[1] Mubashir M., Shao L., Seed L. (2013). A survey on fall detection: Principles and approaches. Neurocomputing.

[2] Yang L., Ren Y., Hu H., Tian B. (2015). New fast fall detection method based on spatio-temporal context tracking of head by using depth images. Sensors.

[3] Desai K., Mane P., Dsilva M., Zare A., Shingala P., Ambawade D. A novel machine learning based wearable belt for fall detection. Proceedings of the 2020 IEEE International Conference on Computing, Power and Communication Technologies (GUCON).

[4] Rougier C., Meunier J., St-Arnaud A., Rousseau J. (2011). Robust video surveillance for fall detection based on human shape deformation. IEEE Trans. Circuits Syst. Video Technol..

[5] Singh A., Rehman S.U., Yongchareon S., Chong P.H.J. (2020). Sensor technologies for fall detection systems: A review. IEEE Sens. J..

[6] Erol B., Amin M.G. Fall motion detection using combined range and Doppler features. Proceedings of the 2016 24th European Signal Processing Conference (EUSIPCO).

[7] Zhang R., Cheng L., Wang S., Lou Y., Gao Y., Wu W., Ng D.W.K. (2024). Integrated sensing and communication with massive MIMO: A unified tensor approach for channel and target parameter estimation. IEEE Trans. Wirel. Commun..

[8] Ma Y., Miao C., Long W., Zhang R., Chen Q., Zhang J., Wu W. (2024). Time-Modulated Arrays in Scanning Mode Using Wideband Signals for Range-Doppler Estimation With Time-Frequency Filtering and Fusion. IEEE Trans. Aerosp. Electron. Syst..

[9] Hu Y., Zhang F., Wu C., Wang B., Liu K.R. (2021). DeFall: Environment-independent passive fall detection using WiFi. IEEE Internet Things J..

[10] Mastorakis G., Makris D. (2014). Fall detection system using Kinect’s infrared sensor. J. Real-Time Image Process..

[11] Erol B., Amin M.G. (2019). Radar data cube processing for human activity recognition using multisubspace learning. IEEE Trans. Aerosp. Electron. Syst..

[12] Ogawa Y., Naito K. Fall detection scheme based on temperature distribution with IR array sensor. Proceedings of the 2020 IEEE International Conference on Consumer Electronics (ICCE).

[13] Qiao X., Feng Y., Liu S., Shan T., Tao R. (2022). Radar point clouds processing for human activity classification using convolutional multilinear subspace learning. IEEE Trans. Geosci. Remote Sens..

[14] He M., Nian Y., Zhang Z., Liu X., Hu H. Human fall detection based on machine learning using a THz radar system. Proceedings of the 2019 IEEE Radar Conference (RadarConf).

[15] Wang B., Guo L., Zhang H., Guo Y.X. (2020). A millimetre-wave radar-based fall detection method using line kernel convolutional neural network. IEEE Sens. J..

[16] Trange A. (2021). FMCW mmWave Radar for Detection of Pulse, Breathing and Fall within Home Care. Master of Science Thesis.

[17] Anishchenko L., Zhuravlev A., Chizh M. (2019). Fall detection using multiple bioradars and convolutional neural networks. Sensors.

[18] Feng X., Shan Z., Zhao Z., Xu Z., Zhang T., Zhou Z., Deng B., Guan Z. (2023). Millimeter-Wave Radar Monitoring for Elder’s Fall Based on Multi-View Parameter Fusion Estimation and Recognition. Remote Sens..

[19] Jokanović B., Amin M. (2017). Fall detection using deep learning in range-Doppler radars. IEEE Trans. Aerosp. Electron. Syst..

[20] Chen V.C., Li F., Ho S.S., Wechsler H. (2006). Micro-Doppler effect in radar: Phenomenon, model, and simulation study. IEEE Trans. Aerosp. Electron. Syst..

[21] Patole S.M., Torlak M., Wang D., Ali M. (2017). Automotive radars: A review of signal processing techniques. IEEE Signal Process. Mag..

[22] Helen Victoria A., Maragatham G.J.W.N. (2021). Activity recognition of FMCW radar human signatures using tower convolutional neural networks. Wirel. Netw..

[23] Clemente C., Pallotta L., De Maio A., Soraghan J.J., Farina A. (2015). A novel algorithm for radar classification based on Doppler characteristics exploiting orthogonal pseudo-Zernike polynomials. IEEE Trans. Aerosp. Electron. Syst..

[24] Clemente C., Balleri A., Woodbridge K., Soraghan J.J. (2013). Developments in target micro-Doppler signatures analysis: Radar imaging, ultrasound and through-the-wall radar. EURASIP J. Adv. Signal Process..

[25] Li X., He Y., Jing X. (2019). A survey of deep learning-based human activity recognition in radar. Remote Sens..

[26] Gurbuz S.Z., Amin M.G. (2019). Radar-based human-motion recognition with deep learning: Promising applications for indoor monitoring. IEEE Signal Process. Mag..

[27] Stadelmayer T., Santra A., Weigel R., Lurz F. (2021). Data-driven radar processing using a parametric convolutional neural network for human activity classification. IEEE Sens. J..

[28] Simonyan K., Zisserman A. (2014). Very deep convolutional networks for large-scale image recognition. arXiv.

[29] Graves A., Schmidhuber J. (2005). Framewise phoneme classification with bidirectional LSTM and other neural network architectures. Neural Netw..

[30] Hochreiter S., Schmidhuber J. (1997). Long short-term memory. Neural Comput..

[31] Ullah A., Muhammad K., Del Ser J., Baik S.W., de Albuquerque V.H.C. (2018). Activity recognition using temporal optical flow convolutional features and multilayer LSTM. IEEE Trans. Ind. Electron..

[32] Sadreazami H., Bolic M., Rajan S. (2021). Contactless fall detection using time-frequency analysis and convolutional neural networks. IEEE Trans. Ind. Inform..

